# The effect of whole-body cooling on renal function in post-cardiac arrest patients

**DOI:** 10.1186/s12882-017-0780-6

**Published:** 2017-12-29

**Authors:** Silvia De Rosa, Massimo De Cal, Michael Joannidis, Gianluca Villa, Jose Luis Salas Pacheco, Grazia Maria Virzì, Sara Samoni, Fiorella D’ippoliti, Stefano Marcante, Federico Visconti, Antonella Lampariello, Marina Zannato, Silvio Marafon, Raffaele Bonato, Claudio Ronco

**Affiliations:** 1 International Renal Research Institute of Vicenza (IRRIV), Vicenza, Italy; 2 Department of Nephrology, San Bortolo Hospital, Vicenza, Italy; 3 Department of Anesthesia and Intensive Care, San Bortolo Hospital, Viale Rodolfi 37, 36100 Vicenza, Italy; 4 Division of Intensive Care and Emergency Medicine, Department of Internal Medicine, Medical University Innsbruck, Innsbruck, Austria; 5 Department of Health Science, Section of Anaesthesiology and Intensive Care, University of Florence, Florence, Italy; 6 Instituto Nacional de Cardiología-Ignacio Chávez, Mexico City, Mexico

**Keywords:** Acute kidney injury, Cardiac arrest, Ischemia reperfusion injury, Hypothermia, Rewarming Injury

## Abstract

**Background:**

To evaluate the incidence of Acute Kidney Injury (AKI) during therapeutic hypothermia (TH) and rewarming in comatose patients resuscitated from Cardiac Arrest (CA).

**Methods:**

We have performed a pilot study of consecutive comatose patients resuscitated from CA and admitted to our Intensive Care Unit (ICU) from January 2013 to March 2015. The surface cooling devices used were: 1) Arctic Sun® 5000; 2) Blanketrol® III. Data obtained at baseline and during TH included: temperature trend and rate, serum creatinine, interleukin 1-beta, interleukin 6 (IL-6), urinary Interleukin-18 (uIL-18), diuretic use, urine output, fluid balance (FB). AKI was defined according to Kidney Diseases Improving Global Outcomes (KDIGO) criteria.

**Results:**

Thirty-six patients were treated with TH out of 46 ICU admissions (78%). According to KDIGO classification, 21 (58%) had no evidence of AKI while 15 (41.7%) presented AKI during TH. In particular, the incidence of AKI was 2.8% at 24 h, 33.33% at 48 h and 30.6% at 72 h from the onset of cooling. Slower rewarming (above 600 min) was associated with with a non-significant lower incidence of AKI and with a non-significant lower levels of IL-6 and IL-18u. Only two patients required renal replacement therapy during TH (7.6%). Median cumulative FB was 2441 [437–4043] ml for all patients; 3140 [1421–4347] and 1332 [−131–3772] specifically for AKI and not-AKI patients.

**Conclusions:**

The hypothermia treatment, if not well performed, could be a double-edged sword for kidneys: whereas hypothermia may confer protection by reducing metabolism and oxygen consumption, rapid rewarming could nullify benefits leading to a worsening of kidney function and AKI. Additional clinical studies are needed to determine the optimal rewarming rate and strategy.

## Background

Cardiac Arrest (CA) and resuscitation are an example of a whole-body Ischemia/Reperfusion (I/R) injury characterized by a multi-organ dysfunction and systemic activation of the innate immune system causing a ‘sepsis-like syndrome’ [1, 2]. The restoration of spontaneous circulation (ROSC) after prolonged, complete, whole-body ischemia is an unnatural pathophysiological state created by successful cardiopulmonary resuscitation (CPR) [1]. The complex phase of resuscitation which begins when patients regain spontaneous circulation after CA has been defined by the International Liaison Committee on Resuscitation (ILCOR) as “post–cardiac arrest syndrome” [1]. The ischemia and reperfusion during CA, resuscitation and post-resuscitation phases can particularly affect the kidney: I/R injury leads to damage of epithelial cells by increased oxygen radical production [2] and peroxidation of lipids [3, 4] but also to interstitial inflammation and interstitial “micro-vasculopathy” that are involved in abnormal repair processes including incomplete repair of tubular cell and fibrosis development [4]. The cross-talk between the kidney and peripheral immune system in humans after whole-body I/R injury and the changes over time in the inflammatory response are speculative [5] and aside from cardiogenic shock limited information are available regarding predisposing factors and outcome of acute kidney injury (AKI) after CA [6, 7]. Although therapeutic hypothermia (TH), also termed targeted temperature management, is an established intervention (ILCOR/American Heart Associatio) used for neuroprotection [3, 4] though recent data challenge the benefit of TH using target temperature lower than 36 °C [8, 9]. The effect of TH on renal protection is uncertain and poorly investigated.

The purpose of this study was to investigate the effect of TH on the development of AKI and on renal outcomes in comatose patients resuscitated from CA and treated with surface cooling techniques. We hypothesized that TH could be a protective strategy against renal I/R injury while the shift from hypo- to normothermia may cause rewarming injury.

## Methods

### Study design

This was a single center observational study. The Institutional Review Board approved the protocol and written informed consent was obtained from the nearest relatives. Data were collected prospectively on 36 patients between January 2013 and March 2015.

### Patients selection criteria

All patients admitted to the emergency department for out-of-hospital CA and treated with TH in the intensive care unit (ICU) with a standardized protocol [10] were considered eligible for the study. Inclusion criteria were: 18 years or older post-cardiac arrest patients (asystole or pulseless electrical activity or ventricular fibrillation or non-perfusing ventricular tachycardia as initial rhythm) and ST-segment elevation myocardial infarction. Exclusion criteria were: time from ROSC to initiation of cooling >6 h; body temperature below than 30 °C on admission; major trauma; severe burns; major surgery less than 72 h; sepsis and septic shock; active bleeding or known pre-existing coagulopathy; intracranial bleeding; pregnancy; aggressive care. Patients were treated either by the Arctic Sun® or standard cooling blankets Blanketrol® IIIas decided by the treating physician. The time between collapse and start of cooling was calculated based on data provided by the paramedic team.

### Patient management

In all patients, the resuscitation attempt followed the European Resuscitation Council 2010 guidelines for basic and advanced cardiac life support and post-resuscitation care [11]. In conjunction with resuscitation procedures, the patient was assessed for study inclusion. ROSC was defined as an organized rhythm and palpable pulse sustained for at least 20 min. In pre-hospital setting, infusion of ice-cold normal saline, the use of cold packs or the trans-nasal cooling was permitted and it was according to choose of the physician involved in the first aid. If necessary, a coronary angiogram and a percutaneous coronary intervention were performed before the admission to ICU. Once the patient fulfilled the inclusion criteria and in agreement with our protocol, sedation, analgesia, and paralysis were started with: a Propofol bolus of 2 mg/kg, followed by continuous infusion (0.5–3 mg/Kg/h) or Midazolam bolus of 0.03–0.04 mg/kg, followed by continuous infusion (0.2 mg/kg/hr); continuous infusion of Remifentanil (0.02–0.2 mcg/kg/min) and of Cisatracurium continuous infusion of 1 mcg/kg/min. Other treatments such as magnesium, fluids, and vasopressors were left to the discretion of treating physicians. All patients had a bladder temperature probe inserted for a continuous measurement of core temperature, which provides feedback to the cooling devices, freely chosen by the physician involved in the treatment. The cooling devices used were:

1. Blanketrol® III, a cold blanket wrapped around the upper torso. Automatic control mode, designed to limit the magnitude of the difference between the bladder and circulating water temperature (gradient modes), was set with a bladder temperature of 33 °C; If the patient’s temperature was lower than the target temperature, the device heated the circulating water to the highest allowable water temperature (42 °C); on the contrary, if the temperature was higher than the target temperature, the device cooled the circulating water to the lowest allowable water temperature (4 °C) until the achieving the body target temperature. The circulation of the water without heating or cooling characterizes the reach of the target temperature.
2. Arctic Sun® 5000, the cooling pads applied on the back, chest and thighs. Automatic mode was set to a target temperature of 33 °C, and the maximum cooling rate was used. The decision of the cooling device was performed by treating physician based on his preference. Target temperature was achieved and maintained for 24 h. At the end of the maintenance phase, rewarming phase started with a target temperature of 36 °C and with a rewarming rate between 0.25–0.5 °C per hour. Once the patient’s temperature reached 36 °C, the device was turned off.

### Data collection and blood sampling

Demographic variables and pre-hospital data were collected prospectively for all patients at the admission utilizing the Utstein criteria. Hemodynamic parameters and temperature were measured continuously. Data obtained on admission and 6, 24, 48 and 72 h included: temperature trend and rate, serum creatinine (sCr), interleukin 6 (IL-6), interleukin 1-beta (IL-1beta), urinary interleukin −18 (uIL-18), urine output, fluid balance.

AKI was defined according to Kidney Diseases Improving Global Outcomes (KDIGO) criteria [12, 13]. Estimated Glomerular Filtration Rate and urinary output criteria were not used for AKI diagnosis and staging in this study. Baseline sCr was calculated using the Modification of Diet in Renal Disease (MDRD) equation (back-estimation). In particular, in absence of previous values, the baseline creatinine has been calculated as follow: GFR(Glomerular Filtration Rate) = (75/[186 × (age^-0.203^) × (0.742 if female) × (1.21 if black)])^-0.887^). For each time point, sCr considered for AKI diagnosis was corrected for fluid balance according to recent evidence [14]. The Sequential Organ Failure Assessment (SOFA) score was calculated using standard methods. Quantitative determination of uIL-18 was performed by Human Instant ELISA kit (eBioscience, San Diego, CA, USA). Cytokines determinations were performed according to manufacturer’s protocol and instructions. Optical density was read by using a VICTORX4 Multilabel Plate Reader (PerkinElmer Life Sciences, Waltham, MA, USA) at 450 nm. The concentration values for these molecules were calculated from standard curves, according to the manufacturer’s protocols. All tests were performed in triplicate.

### Outcomes

The primary outcome was the development of AKI during all treatment period in patients treated with either one of the two cooling devices. The secondary outcomes were: the evaluation of association between duration of each phase of TH (i.e. cooling, hypothermia, rewarming) and AKI; the variation of sCr, urine output, fluid balance and serum cytokine during TH; the effects of rewarming phase on development of AKI. The outcomes were assessed by study personnel who were aware of the type of external cooling assigned for the subject.

### Statistical analysis

The Shapiro-Wilk test was used to test the data for normality. The differences between AKI and not-AKI patients were tested using Mann-Whitney-Wilcoxon for continuous variables, according to the not-normal distribution of data, and presented as median [I-III interquartile]. Categorical variables were analyzed using the chi-square test and given as a percentage. Differences between AKI and not-AKI have also been evaluated through univariate and multivariate analysis; results are presented in tables as *p* values, Odds ratio (OR) and 95% confidence interval (95% CI). A *p* value less than 0.05 was considered for statistical significance. Data were analyzed using STATA 12 software (STATA corp, 490, Lakeway Drive College Station, 77845, Texas, US).

## Results

### Baseline demographics

**Fig. 1:**
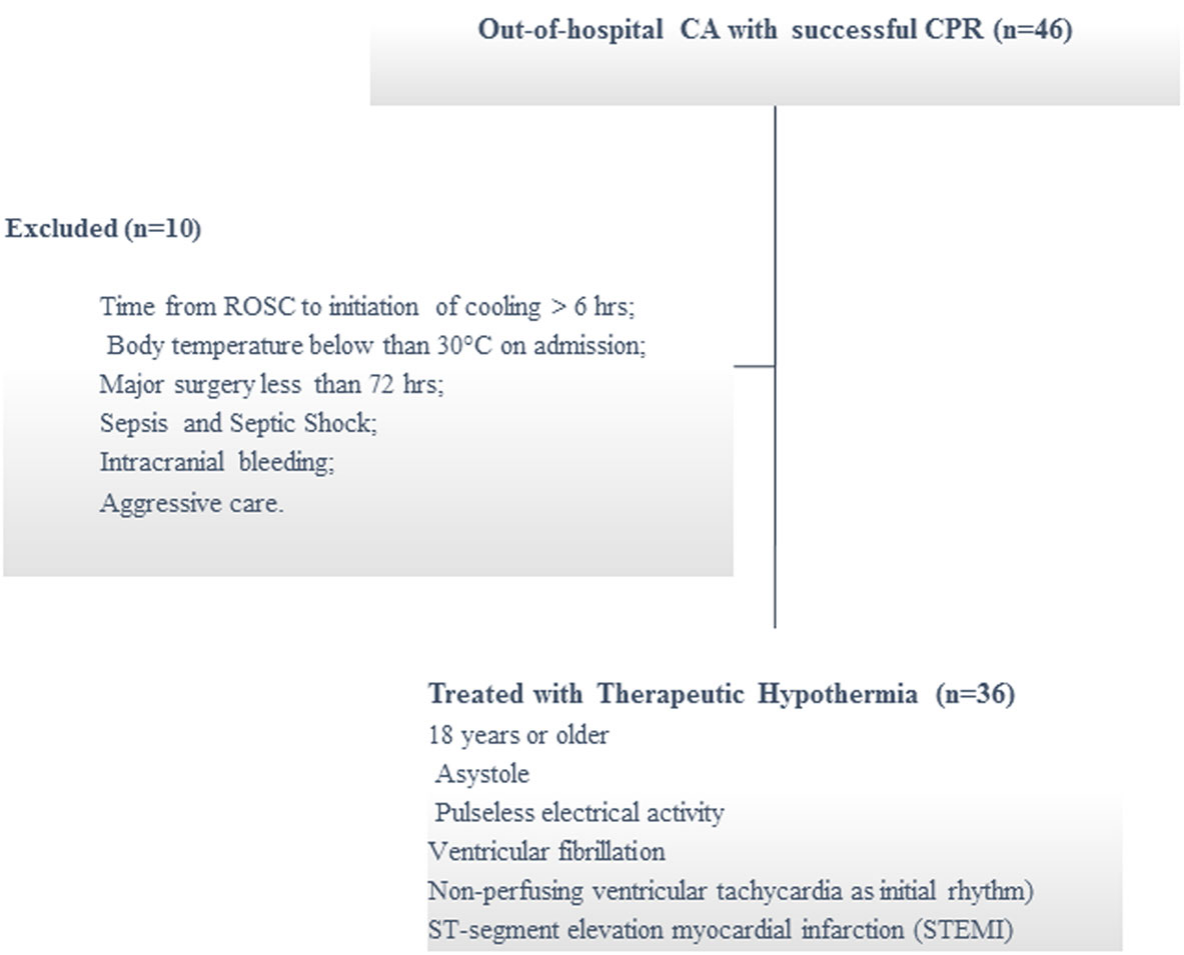
Patient enrollment flow chart

From January 2013 till March 2015, 36 patients with successful CPR were treated by TH out of 46 ICU admissions for CA (78%) to the Department of Anesthesia and Intensive Care Medicine, San Bortolo Hospital (Fig. 1) and followed for the development of AKI during ICU stay (Table 1). Ten (28%) patients were treated with Blanketroll III (Blanket Group), and twenty-six (72%) patients were treated with the Arctic Sun Cooling Machine (Artic Sun Group).

**Table 1:** Baseline demographic data, resuscitation details and comorbidities

	Entire Cohort *n* = 36	AKI *n* = 15	No AKI *n* = 21	*p*	OR	95% CI
Gender				0.94	0.94	0.17–4.99
Male	29 (80.5%)	12 (80%)	17 (81%)			
Female	7 (19.5%)	3 (20%)	4 (19%)			
Age (yrs)	60.5 [53–72.5]	60 [52–77]	63 [54–67]	0.5	1.02	0.96–1.07
BMI	26 [23–28.7]	27.6 [23.2–29.4]	24.7 [23–27.8]	0.18	1.13	0.94–1.35
SOFA score	9 [8–11]	10 [8–11]	9 [8–11]	0.79	105	0.75–1.46
APACHE II score	24 [21–26]	26 [22–29]	24 [21–24]	0.04	1.25	1.02–1.55
Presenting Cardiac Rhythm				0.95	1.05	0.20–5.43
VF/VT	29 (81%)	12 (80%)	17 (81%)			
PEA/Asystolia	7 (19%)	3 (20%)	4 (19%)			
Prehospital cooling	11 (30.5%)	6 (40%)	5 (23.8%)	0.12	0.35	0.09–1.30
Re-arrest after ROSC				0.68	0.84	0.371.91
Shockable	11 (30.5%)	6 (40%)	5 (23.8%)			
No-Shockable	1 (2.8%)	0 (0%)	1 (4.8%)			
None re-arrest	24 (66.6%)	9 (60%)	15 (75%)			
ECM				0.24	1.58	0.74–3.39
Trained	11 (30.6%)	3 (20%)	8 (38.1%)			
Untrained	12 (33.3%)	5 (33.3%)	7 (33.3%)			
Lukas	5 (13.9%)	3 (20%)	2 (9.5%)			
Non performed	8 (22.2%)	4 (26.7%)	4 (19%)			
Total Number Of Shock	3 [1.2–7]	3 [2–4]	3 [1–7]	0.39	086	0.61–1.21
Total Adrenaline Given (mg)	1 [0–3.75]	0 [0–3]	1 [0–4]	0.64	0.94	0.71–1.23
Total Amiodarone Given (mg)	0 [0–300]	0 [0–0]	0 [0–300]	–	–	–
Comorbidities						
Hypertension	14 (22.2%)	8 (38.1%)	6 (28.6%)	0.14	0.35	0.09–1.40
Previous Kidney Disease	0 (0%)	0 (0%)	0 (0%)	–	–	–
Previous Cardiac Surgery	2 (5.5%)	2 (13.3%)	0 (0%)	–	–	–
Coronary Artery Disease	24 (66.6%)	11 (73.3%)	13 (61.9%)	0.48	0.59	0.14–2.50
Insulin Required Diabetes	8 (22%)	7 (46.7%)	1 (4.7%)	0.01	0.05	0.005–0.48
COPD	3 (8.4%)	3 (20%)	0 (0%)	–	–	–

All quantitative variables are presented as median value [IQR], while quantitative data as total number (%). The differences between AKI and No-AKI patients has been tested through multivariate analysis

*Abbreviations*: *BMI* Body Mass Index, *SOFA* Sequential Organ Failure Assessment, *APACHE* Acute Physiology and Chronic Health Evaluation, *VF* Ventricular Fibrillation, *VT* Ventricular Tachycardia, *PEA* Pulseless Electrical Activity, *ROSC* Restoration Of Spontaneous Circulation, *ECM* External Cardiac Massage, *COPD* Chronic Obstructive Pulmonary Disease, *AKI* Acute Kidney Injury

### Temperature trend and duration of each treatment phase

At the initiation of cooling, the temperature dropped from a median pre-treatment temperature of 35.6 [34.7–36] °C for Artic Sun Group and 35.7 [35.1–36.1] °C for Blanket Group, to respectively a median plateau value of 33 °C and 33.1 °C. At 6 h, the target temperature of 33 °C was reached in both groups.

**Fig. 2:**
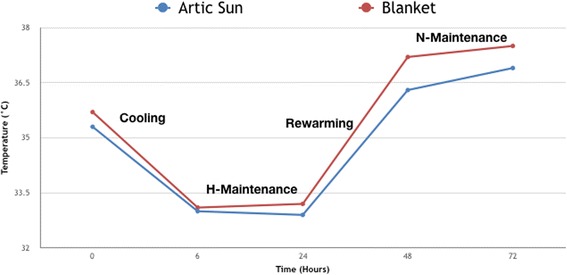
Bladder temperature trend in the Artic Sun Group and Blanket Group within the first 72 h. Data are given as median values

At 24 h, the median temperature reached was 32.9 [32.7–33] °C in the Artic Sun Group and 33.3 [32.9–33.5] °C in the Blanket Group. The median temperature kept during the rewarming period was 36.5 [36–36.7] °C in the Artic Sun Group and 37.5 [36.8–37.7] °C in the other Group, respectively. The median temperature kept during the normothermic period was 36.7 [36.2–37] °C in the Artic Sun and 37.4 [37.2–37.8] °C in the other Group (Fig. 2). The duration of each phase and the time to fever, defined as the temperature above a threshold of 37.5 °C, is expressed in Table 2.

**Table 2:** Renal outcomes

	Entire Cohort *n* = 36	AKI *n* = 15	No AKI *n* = 21	*p*	OR	95% CI
LVEF <35	14 (38%)	7 (46.7%)	7 (33.3%)	0.37	0.53	0.13–2.14
VIS						
Admission	0 [0–5]	0 [0–0]	0 [0–0]	0.75	1.02	0.89–1.16
6 h	5.7 [0–16]	3.7 [0–8.9]	9.8 [0–16.6]	0.36	0.95	0.85–1.05
24 h	5.5 [2.1–11]	4 [2.4–6.5]	7 [2–12.3]	0.27	0.91	0.76–1.08
48 h	2 [0–8.9]	3.9 [0–11]	2.1 [0–7]	0.48	0.96	0.85–1.07
72 h	0 [0–5.7]	2.3 [0–6.8]	0 [0–5]	0.15	1.25	0.92–1.72
MAP						
Admission	86 [70–109]	84 [67–112]	89 [71–109]	0.26	0.97	0.93–1.02
6 h	84 [70–100]	82 [70–100]	85 [69–100]	0.56	1.02	0.95–1.09
24 h	83 [71–90]	79 [68–86]	84 [72–92]	0.59	0.98	0.91–105
48 h	82 [69–94]	88 [77–95]	78 [68–89]	0.37	1.03	0.97–1.09
72 h	84 [71–95]	74 [66–90]	92 [82–107]	0.06	0.95	0.89–1.00
Fluid Balance						
24 h	333 [−434–1280]	448 [95–1250]	5 [−703–1477]	0.53	0.99	0.99–1.00
48 h	1522 [488–2236]	1290 [344–2621]	1592 [587–2099]	0.56	0.99	0.99–1.00
72 h	621 [−389–1749]	1155 [129–2168]	393 [−1567–1475]	0.67	0.99	0.99–1.00
Cumulative	2441 [437–4043]	314 [1421–4347]	1332 [−131–3772]	0.54	0.99	0.99–1.00
Urinary Output						
24 h	2242 [1403–2844]	1890 [1100–2853]	2245 [1720–2820]	0.40	0.99	0.99–1.00
48 h	2877 [2362–3552]	2810 [2280–3400]	2990 [2400–3620]	0.19	1.00	0.99–1.00
72 h	3185 [2538–4060]	3010 [2370–4470]	3627 [2605–4040]	0.27	0.99	0.99–1.00
Ventilation days	8 [4–12.7]	6 [3–11]	8 [4–14]	0.94	0.99	0.93–1.07
ICU days	9 [4.5–12.5]	8 [4–12]	9 [4.5–14]	0.99	1.00	0.93–1.07
ICU mortality	23 (63.9%)	9 (60%)	14 (66.7%)	0.68	0.75	0.19–2.96
Hospital mortality	25 (69.4%)	10 (66.7%)	15 (71.4%)	0.99	1	0.93–1.08
90-days mortality	25 (69.4%)	10 (66.7%)	15 (71.4%)	0.99	1	0.93–1.08

Continuous variables are expressed as median [IQR], qualitative data as number (%)

*Abbreviation*: *AKI* acute kidney injury, *ICU* intensive care unit, *LVEF* Left ventricular ejection fraction, *MAP* mean arterial pressure, *RRT* renal replacement therapy, *VIS* vasoactive inotropic score

### AKI development during TH treatment

**Fig. 3:**
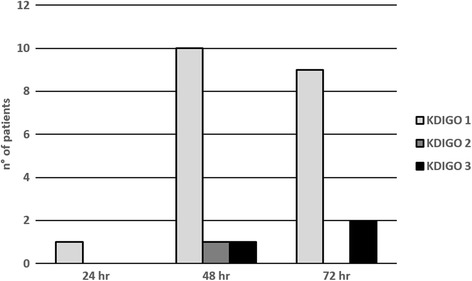
Incidence of AKI at different time points. From 24 h until 48 h there is an increase of AKI patients at KDIGO stage 1 and from 48 h until 72 h there an increase of patients at KDIGO stage 3

According to KDIGO classification, among all 36 patients, 21(58%) had no evidence of AKI while 15(41.7%) had AKI during TH. The incidence of AKI for each time point is reported in Fig. 3.

In all population, the median time for rewarming was 600 [480–720] min. Although there is no statistically significant difference, patients with AKI had a slightly increase in rewarming time compared to patients without AKI, respectively 540 [480–720] min vs 630 [480–720] min (*p* = 0.13) (Table 3).

**Table 3:** Time duration of each treatment phase

	Entire Cohort *n* = 36	AKI *n* = 15	No AKI *n* = 21	*p*	OR	95% CI
Time from CA to 33 °C	280.5 [240–385]	256.5 [216–325]	330.5 [254–390]	0.09	0.96	0.92–1.00
Time from TH induction to 33 °C	184.5 [120–248]	180 [120–210]	192 [120–252]	0.09	1.04	0.99–109
Time of TH duration	1440 [1440–1557]	1440 [1440–1620]	1440 [1440–1500]	0.33	0.99	0.97–1.00
Time from 33 °C to 36 °C	600 [480–720]	540 [480–720]	630 [480–720]	0.13	0.99	0.99–1.00
Time from 36 °C to fever	1380 [487–3180]	2880 [300–4320]	1260 [495–2880]	0.31	1.00	0.99–100

Median duration of treatment phases for all patients and specifically for AKI and no-AKI patients. All periods of time are expressed in minutes

*Abbreviations*: *CA* Cardiac Arrest, *TH* Therapeutic Hypothermia, *AKI* Acute Kidney Injury

### sCr, fluid balance, urine output, diuretic use and need of renal replacement therapy (RRT) during TH treatment

**Fig. 4:**
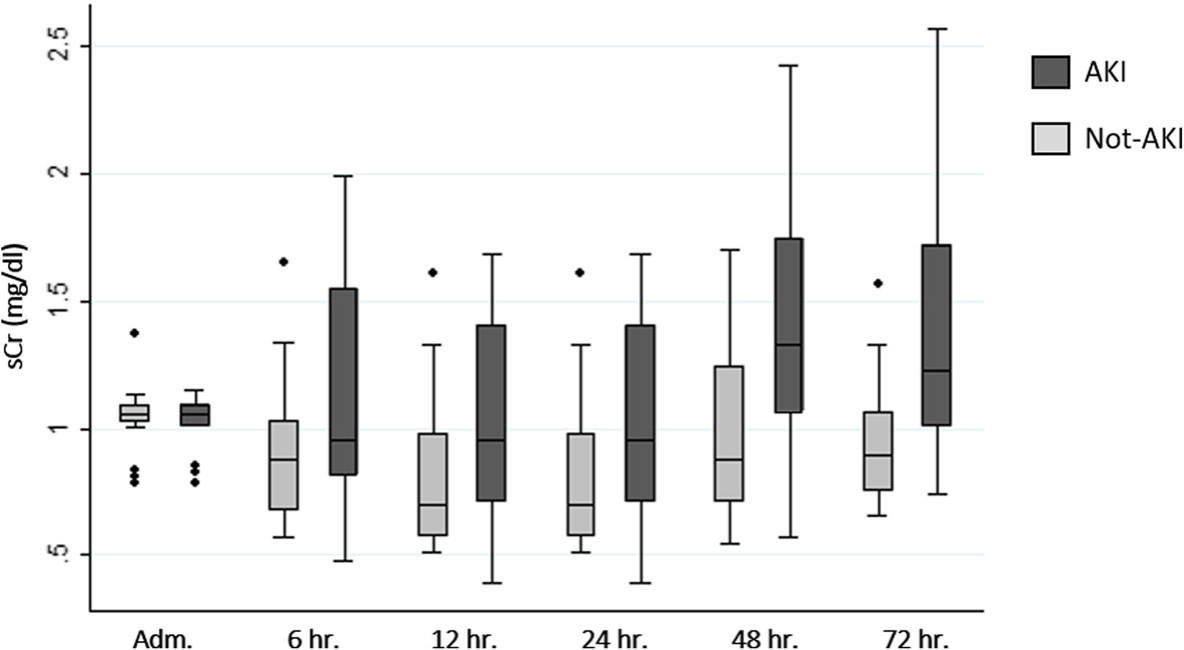
sCr in patients with and without AKI at different time points. sCr levels progressively increases in AKI patients from 24 h until 72 h conversely to non-AKI patients

Considering overall population, median values of sCr based on MDRD formula was 1.06 [1.02–1.09] mg/dl) while sCr at admission was 1.12 [0.94–1.22] mg/dl. These values were: 0.82 [0.63–1.07], 1.04 [0.77–1.32], 1.06 [0.79–1.42] mg/dl respectively at 24 h, 48 h, 72 h. sCr between patients with AKI and no AKI is shown in Fig. 4.

Urine output increased from 24 h until to 72 h. The average daily dose of frusemide was 20 [0–20] mg. N differences have been observed for urinary output between patients treated with or without diuretics at 24 h (*p* = 0.74), 48 h (*p* = 0.35) and 72 h (*p* = 0.23).

In overall population, the median fluid balance from 24 h to 72 h was +2441 [432–4044] ml. Only two patients required RRT during the treatment (7.6%) (Table 2).

### Cytokines concentration during the treatment period

**Fig. 5:**
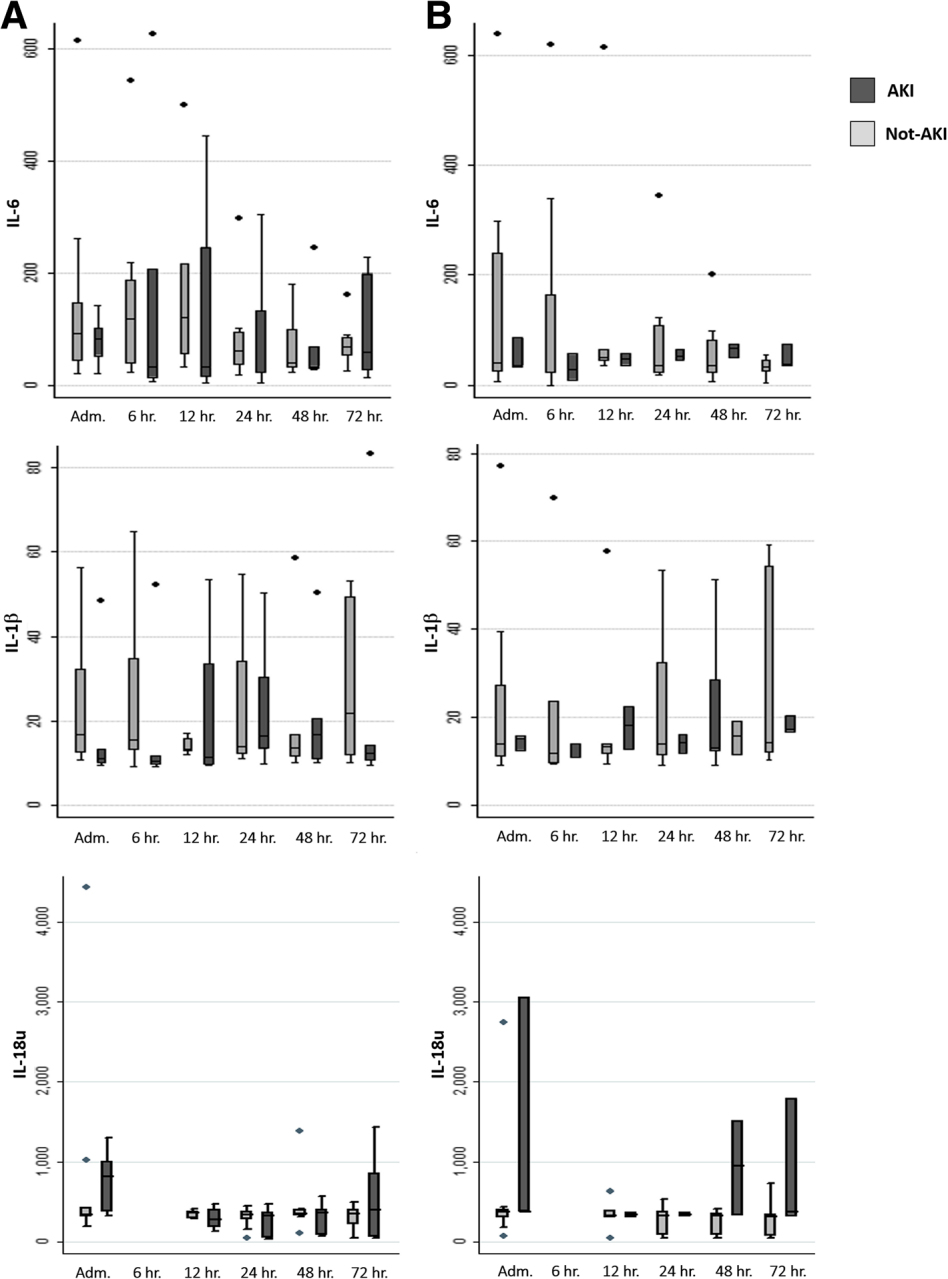
Cytokines in patients with rewarming time below and above 600 min. 5 shows cytokines levels at different timepoint in AKI and not-AKI patients with slow rewarming (Panel **a**) and with rapid rewarming (Panel **b**)

For each time point, the plasmatic concentration of IL-6 and IL-1beta and the urinary concentration of uIL-18 have been described for overall population and for AKI and no AKI patients in Table 4. Figure 5 shows cytokines levels at different timepoint in AKI and not-AKI patients with slow rewarming (Panel A) and with rapid rewarming (Panel B).

**Table 4:** Cytokines trend in the two different groups of patients

	Entire Cohort *n* = 36	AKI *n* = 15	No AKI *n* = 21	*p*	OR	95% CI
IL-6						
Admission	79.05 [31.34–113.52]	82.79 [37.12–93.31]	62.32 [28.66–139.95]	0.28	0.99	0.98–1.00
6 h	56.9 [22.52–165.45]	36.50 [12.1–60.18]	64.96 [22.52–188.4]	0.62	0.99	0.99–1.00
12 h	53.58 [39.04–121.25]	45.17 [28.91–53.76]	63.88 [47.79–177.5]	0.38	0.99	0.99–1.00
24 h	46.75 [22.36–94.37]	46.46 [21.21–64.29]	46.75 [22.69–98.61]	0.80	0.99	0.98–1.00
48 h	44.38 [26.45–78.45]	57.83 [31.93–74.84]	40.63 [24.33–82.06]	0.69	1.00	0.99–1.01
72 h	48.89 [27.85–73.38]	43.47 [27.77–89.84]	48.91 [27.93–67.1]	0.24	1.00	0.99–1.02
IL-1beta						
Admission	13.27 [11.3–20.41]	11.87 [10.87–15.2]	14.91 [12.3–23.22]	0.28	0.96	0.90–1.03
6 h	13.04 [9.93–16.48]	10.76 [9.58–13.98]	14.65 [11.52–20.43]	0.29	0.97	0.91–1.03
12 h	13.13 [11.71–16.54]	12.94 [10.52–20.33]	13.13 [12.34–14.92]	0.74	1.01	0.94–1.08
24 h	14.15 [11.85–21.92]	14.14 [11.43–18.76]	14.79 [11.96–21.92]	0.54	0.98	0.93–1.04
48 h	13.58 [11.62–17.52]	15.93 [11.21–19.01]	13.55 [11.62–16.63]	0.73	0.99	0.93–1.05
72 h	14.21 [11.64–22.27]	14.20 [10.56–17.22]	14.31 [11.64–49.43]	0.51	0.98	0.94–1.03
uIL-18						
Admission	370.13 [319.63–740.5]	559.35 [369.76–997.36]	361.64 [309.94–412.06]	0.29	1.00	0.99–1.00
12 h	325.7 [299.6–375.5]	317.97 [262.60–345.89]	329.16 [303.16–375.9]	0.50	0.99	0.99–1.00
24 h	330.16 [280.93–365.5]	327.42 [288.01–361.53]	330.16 [219.25–373]	0.91	0.99	0.99–1.00
48 h	335.92 [289.68–393.47]	355.5 [301.5–566.52]	331.24 [277.84–365.5]	0.29	1.00	0.99–1.00
72 h	331.96 [207–439.37]	366.61 [331.92–845.5]	323.71 [130.05–405.5]	0.16	1.00	0.99–1.00

All cytokines concentrations are expressed in pg/ml and presented as median value [IQR]. The difference in cytokines concentrations at each time points has been tested again the AKI development through multivariate analysis

*Abbreviations*: *IL-6* Interleukin 6, *IL-1beta* Interleukin 1beta, *uIL-18* urinary Interleukin 18, *AKI*, Acute Kidney Injury

## Discussion

To the best of our knowledge, this is the first study suggesting that the rewarming phase after TH applied to post-CA patients may have an impact on the development of AKI. During the TH period, among all 36 patients, 21 (58%) had no evidence of AKI according to KDIGO classification. AKI is a common occurrence, in particular from out-of-hospital-CA patients. Indeed, post-cardiac arrest is characterized by a whole-body ischemia followed by reperfusion with activation of a systemic inflammatory response and release of injury products into the circulation [5, 6] responsible of additive stimuli for ischemic injury [15]. Following resuscitation from spontaneous CA, AKI is a common complication with increased mortality risk, dialysis requirement and prolonged hospital stay [7, 16, 17]. An intriguing field of research shows the capability of a decrease in whole-body temperature to limit the ischemic injuries. Although TH has been used from 1950s to protect the brain against a global ischemia after CA, there is scant data on the potential benefit of this strategy on kidney endpoints [18, 19]. Experimental studies investigated the effect of body temperature on renal susceptibility. On the basis of renal ischemic preservation by using low body temperature hypothermia, Pelkey et al. demonstrated that minimal temperature changes during renal ischemia alter functional and morphologic outcome [20]. These findings in literature showed that in animals hypothermia is able to reduce the risk of renal failure after renal I/R injury [21, 22]. Renal function is eventually depressed during hypothermia owing to a fall in systemic blood pressure and the effect of hypothermia on organ metabolism itself. As renal blood flow progressively decreases, renal vascular resistance rises, promoting a further decrease in renal flow and a subsequent decrease in glomerular filtration. Increased blood viscosity and vasoconstriction were both responsible for this reduction of flow. In the setting of TH, this effect is maintained for 24 h. In this hypothermic phase, from admission to 24 h, there is a decrease of sCr probably due to decrease in muscle metabolism. Clinical practice is based on sCr measurements to monitor renal function and classify AKI. For this reason, we used a validated criteria to define and classified AKI: KDIGO. It is presumed that TH increases urine output particularly in the induction phase, this phenomenon is called cold-induced diuresis [23]. For this reason, we decide to not consider urinary output criteria for diagnosis of AKI. Our results suggested that in the rewarming phase, which started after 24 h, there is a slight increase of sCr associated with an increase in urine output that did not correlate with diuretic dose. Rewarming is a delicate phase of therapeutic hypothermia. Similar to the brain [24], the rewarming phase seems to be very important for the kidney and we strongly believe that could be dangerous if it is not performed slowly and with adequate time duration. Our results showed that with a rewarming time more than 600 min the risk of AKI is decreased. However, it does not mean that longer rewarming times exclude any risk of AKI. Rewarming of the patient is begun 24 h after the initiation of cooling. The literature recommends rewarming slowly at a temperature of 0.3–0.5 °C every hour. It means that rewarming will take approximately 8 h [25]. Differences in the time of rewarming could impact microinflammation and AKI risk.

In our study, patients who did not develop AKI had median rewarming time of 630 min (10.5 h) whereas those who did develop AKI had a median time of 540 (9 h). Rewarming is a delicate phase of TH. Adverse consequences of rewarming on the whole body may seriously limit the protective effects of hypothermia, leading to secondary injury. In literature, previous studies showed that the rise in body temperature accompanies a vasodilation causing a rewarming shock and then careful fluid monitoring during rewarming is crucial [26]. Probably, controlled rewarming of 0.15 °C per hour should be recommended [27] Our data support this recommendation showing less AKI with rewarming time > 600 min which would translate to a rewarming rate of less than 0,4 °C/h. It should be considered that in patients with severe oliguria or anuria despite volume resuscitation, RRT should be started before rewarming to avoid hyperkalemia or metabolic acidosis due to transcellular electrolyte shifts associated with this phase [28]. Hypothermia suppresses ischemia-induced inflammatory reactions and release of pro-inflammatory cytokines [29]. In our study, we assessed inflammatory response dosing IL-6, IL-1beta and IL-18 and results supported the evidence. In addition, uIL-18, a 22 KDa pro-inflammatory cytokine, produced by immune cells, macrophages and proximal tubular cells, has a role in inflammatory processes that exacerbate renal injury during the extension phase of AKI [30, 31]. This cytokine is an early biomarker for AKI in a variety of clinical scenarios indicating the activation of inflammation in kidney [32–34]. Our results showed that with a rewarming time less than 600 min, there is a more increase in concentration respect to those with a slower rewarming (above 600 min) supporting the hypothesis that rapid rewarming is associated with increase risk of AKI (Table 4). The study has some limitations. First, this is a single center trial with only a relative small number of patients included. Secondly, the patients were assigned to two different cooling methods in a non-randomized fashion. Thus, a bias introduced by the cooling method cannot be excluded. Our results, suggest possible harm by an insufficiently controlled and or too short rewarming period. As feasibility study, there was not a comparison with a normothermic control group. The study is too small to make conclusions about outcomes, although the results are hypothesis generating.

## Conclusions

The hypothermia treatment, if not well performed, could be a double-edged sword for kidneys: whereas hypothermia may confer protection by reducing metabolism and oxygen consumption, rapid rewarming could nullify benefits leading to a worsening of kidney function and AKI. Additional clinical studies are needed to determine the optimal rewarming rate and strategy.

### Key messages

- In our prospective study, a rewarming time of more than 600 min is associated to a decrease of the risk of AKI;
- The uIL-18 levels has a role in inflammatory processes that exacerbate renal injury during the extension phase of AKI. During rewarming time less than 600 min, there is a more increase in concentration respect to those with a slower rewarming (above 600 min) supporting the hypothesis that rapid rewarming is associated with increase risk of AKI.
- Whereas hypothermia may confer protection by reducing metabolism and oxygen consumption, rapid rewarming could nullify benefits leading to a worsening of kidney function and AKI.

## Abbreviations

AKI: Acute Kidney Injury
CA: Cardiac Arrest
CPR: Cardiopulmonary Resuscitatione
GFR: estimated Glomerular Filtration Rate
I/R: Ischemia/Reperfusion
ICU: Intensive Care Unit
IL-1beta: Interleukin 1 beta
IL-6: Interleukin 6
ILCOR: International Liaison Committee on Resuscitation
KDIGO: Kidney Diseases Improving Global Outcomes
MDRD: Modification of Diet in Renal Disease
ROSC: Restoration of spontaneous circulations
Cr: Serum Creatinine
SOFA: Sequential Organ Failure Assessment
STEMI: ST-Segment Elevation Myocardial Infarction
TH: Therapeutic Hypothermiau
IL18: Urinary Interleukin 18
